# Use of a Retrospective Methodology to Examine the Process of Care Surrounding Serious Medical Events in HIV-Positive Patients: A Feasibility Study

**DOI:** 10.1177/2325958219868747

**Published:** 2019-09-04

**Authors:** Erica R M Pool, Vanessa Cooper, Elaney Youssef, Juliet Wright, Jordan Skittrall, Ola Blach, Martin Fisher, Helen Smith

**Affiliations:** 1 University College London, Mortimer Market Centre, London, United Kingdom; 2 Brighton and Sussex University NHS Trust, Elton John Centre, Brighton, United Kingdom; 3 Brighton and Sussex Medical School, University of Brighton, Falmer, Brighton, United Kingdom; 4 Cambridge University Hospitals NHS Foundation Trust, Cambridge, United Kingdom

**Keywords:** HIV, aging, comorbidity, feasibility, missed opportunity

## Abstract

**Introduction:**

Comorbidities are increasingly common among people living with HIV (PLWH) as they age. There is no evidence regarding models of care. We aimed to assess feasibility of a novel methodology to investigate care processes for serious medical events in PLWH.

**Method:**

The method was based on the National Confidential Enquiry into Patient Outcome and Death (NCEPOD). Data were extracted from medical records and questionnaires completed by general practitioners (GPs), HIV physicians, and non-HIV specialist physicians. A panel reviewed anonymized cases and gave feedback on the review process.

**Results:**

Eleven of 13 patients consented to the study. Questionnaires were completed by 64% of HIV physicians, 67% of non-HIV specialist physicians, and 55% of GPs. The independent review panel (IRP) advised improvement in the methodology including data presentation and timing.

**Conclusion:**

This method was acceptable to patients and secondary care physicians. Further work is needed to the improve GP responses and facilitate IRP.

**What Do We Already Know About This Topic?**

HIV services in the United Kingdom were not developed for an aging population of people living with HIV (PLWH), there is an evidence deficit regarding the best model of care for older PLWH with multimorbidity.

**How Does Your Research Contribute to the Field?**

We developed a novel methodology based on NCEPOD to review the current model of care for older PLWH who experience a serious medical event.

**What Are Your Research's Implications Toward Theory, Practice, or Policy?**

The methodology was acceptable to patients and secondary care physicians. However, further research is needed to modify the methodology in order to promote engagement and to identify areas of health care for PLWH who are amenable to change, ensuring that further service design is informed by evidence

## Introduction

As a result of effective antiretroviral therapy (ART), HIV has been transformed into a chronic disease with an excellent prognosis. People living with HIV (PLWH) now have near-normal life expectancy^1^ if ART is initiated promptly and a high level of adherence to treatment is maintained.^2^ Life expectancy is expected to improve even further as patients start therapy earlier in the course of infection and with newer drugs.^2^

The number of older PLWH is rapidly increasing^3^; in 2017, more than one-third of all PLWH in the United Kingdom were aged older than 50 years, compared to just 13% in 2004.^1^ Effective treatment means PLWH now rarely experience opportunistic infections.^4^ However, there are increasing numbers of people with controlled HIV experiencing comorbid illnesses associated with aging, but not traditionally associated with HIV,^5,6^ including cardiovascular, renal, hepatic, bone, and metabolic disorders. Both individual and multiple comorbidities appear to be more common among people with HIV than the general population.^7-14^ It is not yet known to what extent this is caused by HIV, ART, or cofactors such as smoking, alcohol, or recreational drug use, which are all more common among PLWH.^15,16^ Living with multiple comorbidities (multimorbidity) can have wide-reaching implications. In PLWH, multimorbidity has been associated with reduced quality of life,^17^ difficulty maintaining employment,^18^ depression,^19^ increased use of medicines and polypharmacy, health care, hospitalizations, and mortality.^5,20-22^ The prevention, prompt detection, and effective management of serious medical events and related comorbidities is now a priority.

The current cohort of older PLWH with an array of comorbidities is unprecedented, and HIV services in the United Kingdom were not developed with this population in mind. A new approach to care may be required to meet their needs. Over recent years, several new approaches have been proposed to detect and manage comorbidities. These include a wider involvement of primary care in the management of HIV similar to the integrated model that applies to other chronic conditions in the National Health Service (NHS),^17^ combined clinics with 1 or more specialists working alongside the HIV clinician,^18^ HIV specialists adopting a special interest (eg, liver disease),^19^ and dedicated clinics for enhanced screening for comorbidities.^20^ Contrary to the recommendations of the Department of Health,^21^ these new approaches to care have been developed in the absence of an evidence base and without consideration of patients’ preferences.

In order to develop novel and appropriate approaches for care of HIV-positive patients with comorbidities, it is necessary to understand the way care is currently delivered and, more importantly, which processes are amenable to improvement. We have developed a novel methodology, derived from that used by the National Confidential Enquiry into Patient Outcome and Death (NCEPOD), to examine the process of care of HIV-positive patients who had experienced a preventable serious medical event.

### Aim

The aim of this pilot study was to examine the feasibility of using this retrospective methodology. If feasible, this methodology can be used to (1) examine the process of care of HIV-positive patients who have experienced a serious medical event (myocardial infarction [MI], stroke, drug–drug interaction [DDI], or progression to chronic kidney disease [CKD] stage 3); (2) identify aspects of care that could have been provided differently to improve care; and (3) use these insights to make recommendations for a future model of care.

## Method

**Figure 1.**
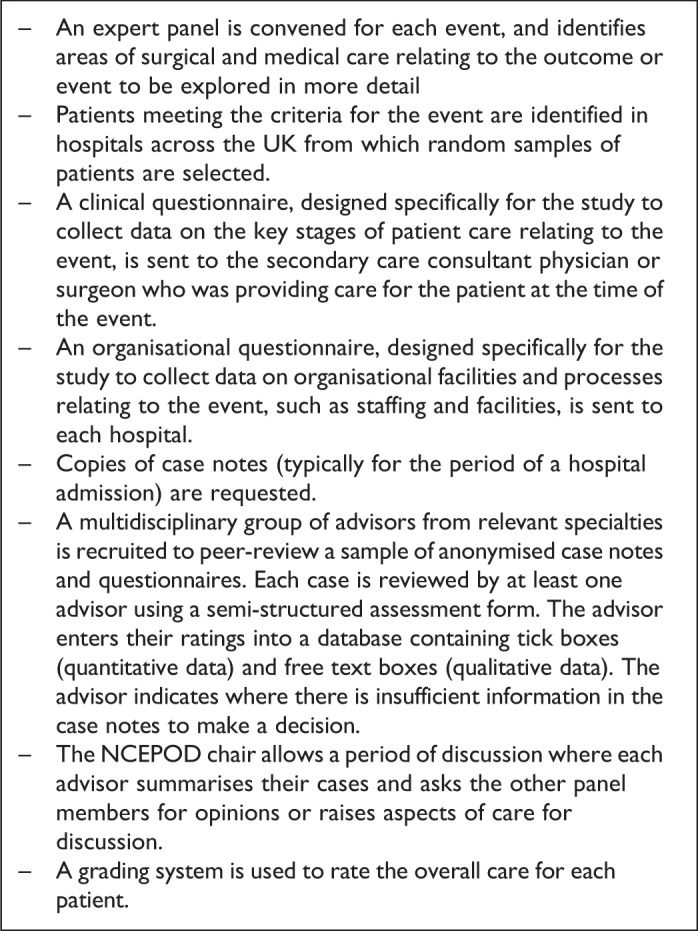
The stages typically followed in a National Confidential Enquiry into Patient Outcome and Death (NCEPOD) study.

The methodology used in this study was based on that used in the United Kingdom by the NCEPOD to identify remediable factors in the process of care of patients who experienced a specific outcome or event NCEPOD methods have been used to investigate a range of clinical events including deaths due to chemotherapy, pain in sickle cell disease, and acute kidney injury, among others.^23^ To our knowledge, NCEPOD methods have not been previously used to investigate events specifically in PLWH. The stages typically followed in an NCEPOD study can be found in Figure 1; they involve review of a sample of cases by a multidisciplinary panel, with a semistructured assessment and a focus on qualitative outcomes.^23^ We adapted this methodology and applied it to examine the process of care of PLWH who had experienced a serious medical event using a 3-stage process:

1. Questionnaire completion by clinicians involved in the case
2. Case note review
3. Review by an independent review panel (IRP).

This study was undertaken at Brighton and Sussex University Hospital from January to August 2015.

### Definition of the Serious Medical Events

The 4 serious medical events were selected on the basis that they were theoretically preventable, and therefore there may have been a missed opportunity in the care of the patient. Those selected were MI, stroke, a serious DDI (prescription of interacting drugs which should not be coprescribed) according to www.hiv-druginteractions.org,^24^ and progression to CKD stage 3 (estimated glomerular filtration rate of 30 to 59 mL/min/1.73 m^2^ for >90 days).^25^

### Participants

**Figure 2.**
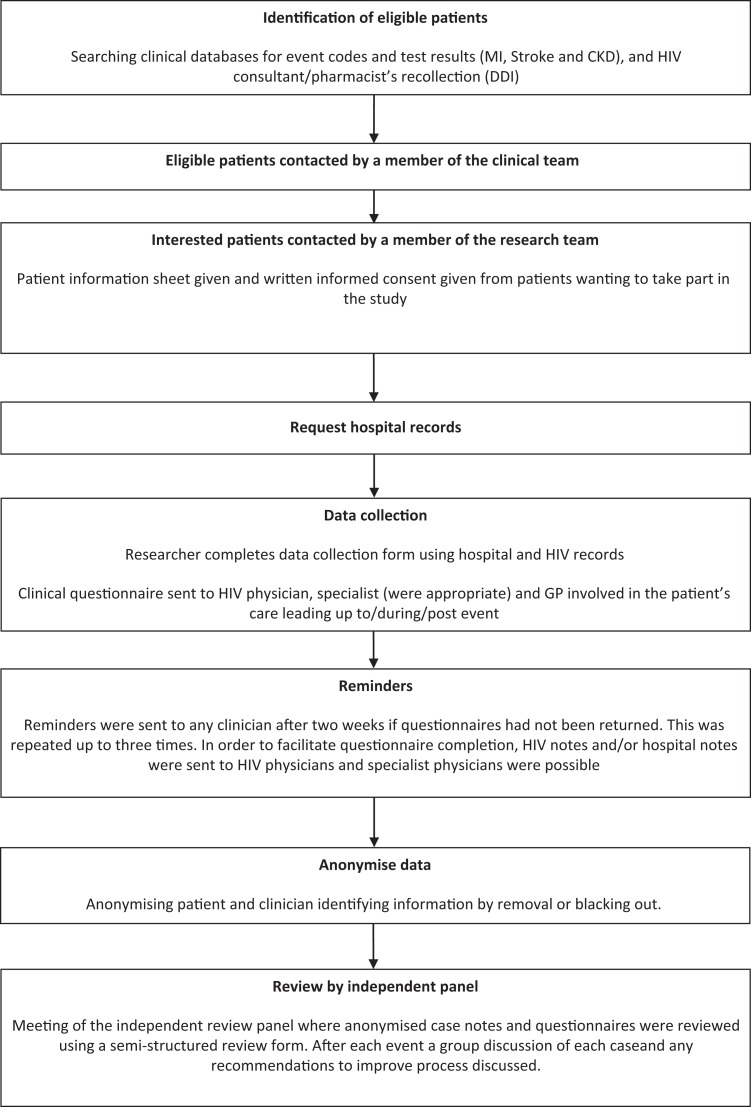
Flow diagram showing the stages of the research.

People living with HIV aged ≥16 years who had experienced MI, stroke, DDI, or progression to CKD stage 3 within the past 3 years were identified by the clinical team at Brighton and Sussex University Hospital (Figure 2). Eligible patients were first approached by their usual clinician, and if interested, a researcher contacted them to provide a patient information sheet and request consent.

### Sample Size

To explore the feasibility of the method, we aimed to recruit a total of 12 patients, 3 patients with 1 of the 4 conditions. This was in accordance with NCEPOD methods that use a sample of cases and do not attempt to survey all cases with the condition or event of interest. The sample size was thought to be pragmatic and sufficient to assess feasibility, in keeping with other feasibility studies.^25-28^

### Questionnaires and Case Note Review

Draft questionnaires were developed based on NCEPOD questionnaires with input from patients and clinicians. These were then reviewed and pretested by an HIV physician, a non-HIV specialist physician, and 2 general practitioners (GPs) who suggested changes including reducing the length of the questionnaire and simplifying wording. The questionnaires had 2 sections. Section 1 explored the clinical details before, during, and after the event, as well as communication between teams, and section 2 explored experience of completing section 1 including process, time, and suitability of questions. One questionnaire per patient was sent to the HIV physician, non-HIV specialist physician, and GP caring for the patient. If there was no reply after 2 weeks, the clinician received a follow-up phone call or an e-mail.

Hospital case notes were reviewed and data recorded on a structured data collection form by research clinicians with experience in infectious diseases. The information extracted via case note review allowed the questionnaires for secondary care clinicians to be shorter than those originally developed, in order to maximize response rate. Case notes were reviewed from 18 months prior the event to 12 months post event. Data collected included patient demographics, clinical data (investigations, diagnoses, and management), and communication between clinical teams. In addition, hospital notes related to the event were copied and anonymized.

### Independent Review Panel

Panel members were asked to review each of the cases using a structured assessment form. The IRP was comprised of an HIV consultant, an HIV specialist nurse, an HIV pharmacist, 2 GPs, and 2 non-HIV specialist physicians (a geriatrician and a nephrologist). Data bundles were collated for each case and provided to the IRP. These included the questionnaires, case note data collection form, and anonymized medical records. The IRP’s feedback was sought on 2 elements: the review process and the cases. Regarding the review process, areas explored included adequacy of information, missing data, unnecessary information, and what could be done differently to improve the review process. Regarding the cases, the process of care surrounding the event was explored; areas included primary and secondary prevention, communication between teams, ownership of care, and an overall rating of care. After completion of the assessment form by the IRP, there was a case discussion in order to capture additional views on the review process. The discussions were recorded and analyzed.

### Outcome Measures

The factors used to assess feasibility were (1) the proportion of eligible patients who gave consent to participate in the study and barriers to consent; (2) the proportion of clinicians contacted who returned completed questionnaires; (3) barriers to questionnaire completion; (4) the quality of data collected by the questionnaires (proportion of items completed and feedback on the process); and (5) feedback from the IRP on the quality of information received and the review process. The secondary outcome was the IRP’s rating of the overall care received by patients who had experienced each event.

### Data Analysis

Data from clinician questionnaires and structured assessment forms completed by the IRP were entered into a Microsoft Excel database. Descriptive data were summarized and presented in tables. Data from free-text boxes and panel discussions were reviewed, and content analysis was undertaken by a researcher (E.Y.).

### Procedures in Place in Case Harmful Practice Was Identified

In advance of commencing the case reviews, procedures that were established in case harmful practice was identified during case review, although no such practice was identified in our study. If harmful practice had been identified, the chief investigator (CI—J.W.) would have accessed a password-protected database and relinked the participant’s unique study code to their name and hospital number and contacted the relevant clinical team. If there had been significant concern that the patient was at ongoing risk, the CI would have contacted the trust medical director (for secondary care) or NHS England (for primary care). If we had identified an event where the duty of candor applied^29,30^ and moderate harm, severe harm, or death occurred, then the CI would have informed the relevant clinical team(s) and deferred the duty of candor to the clinical team(s).

### Ethical Approval and Informed Consent

This study was approved by the Research Ethics Service Committee West Midlands—Coventry and Warwickshire; reference number 15/WM/0039. All patient participants provided written informed consent prior to enrolment in the study. Written information regarding the study was provided to clinicians completing the questionnaires; written consent from the clinicians was not required.

## Results

The primary outcome measures of this study relate to the feasibility of the method, and the secondary outcomes relate to the IRP review of each case.

### Feasibility Outcomes

#### Patient recruitment

**Figure 3.**
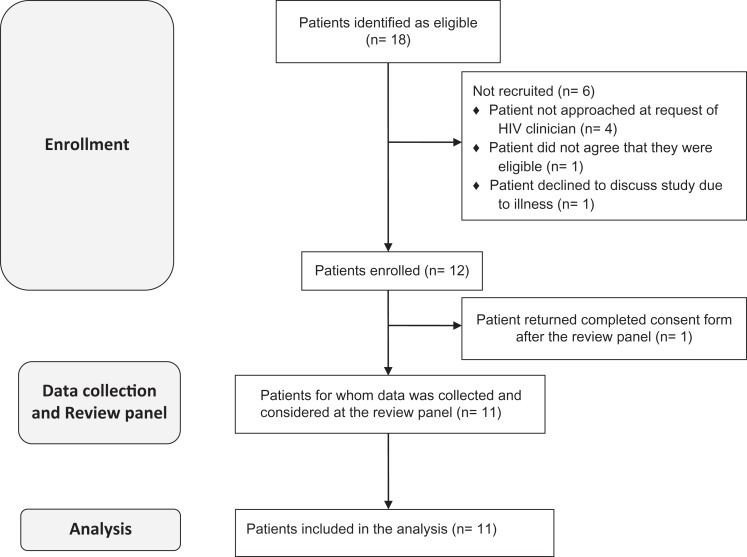
Patient recruitment.

Patient recruitment is summarized in Figure 3. Of 18 patients identified as eligible for the study, 11 (61.1%) patients were recruited. Four patients were not contacted because their HIV physician felt that it would not be appropriate, and 2 patients were contacted but declined to take part. Of the 2 patients who declined, 1 declined due to ill health and 1 queried their eligibility. One patient did not provide written consent until after the review panel meeting had taken place; therefore, no questionnaires or data collections were completed.

#### Questionnaire responses

Overall, 31 questionnaires were sent out relating to the 11 patient cases: 11 to HIV physicians, 11 to GPs, and 9 to non-HIV specialist physicians. In 2 cases, the patient had not seen a non-HIV specialist physician during the study period; therefore, for these cases, questionnaires were only sent to the HIV physician and GP. In total, 17 (54.8%) of 31 questionnaires were returned and completed, and this differed according to the role of the clinician. A similar proportion of questionnaires were returned completed by HIV physicians and non-HIV specialist physicians, 63.6% and 66.7%, respectively. One questionnaire could not be completed, as the only HIV physician involved in the patient’s care died during the study period. One non-HIV specialist physician reported they did not complete the questionnaire due to being unable to trace the medical notes. The response rate for GPs was lower; 4 (36.4%) of 11 questionnaires were returned and complete. One GP reported the questionnaire was too long, and 2 GPs declined as there was no financial reimbursement for their time. The time taken to complete questionnaires varied from a median of 13 minutes for HIV physicians to 35 minutes for GPs (Table 1). The majority of clinicians answered all questions (Table 1).

**Table 1.** Completion of the Clinician Questionnaires.

	Questionnaires Sent, n	Questionnaires Returned, n (%)	Time Taken, minutes, Median (Range)	Questionnaire Items Completed, %, Median (Range)	Case Notes Returned, n (%)
HIV physician	11	7 (63.6)	13 (5-25)	100 (91-100)	7 (63.6)
Non-HIV physician	9	6 (66.7)	7 (5-10)	100 (82-100)	1 (11.1)
GP	11	4 (36.3)	35 (25-40)	100 (22-100)	4 (36.3)

Abbreviation: GP, general practitioners.

#### Questionnaire feedback

Clinicians indicated that the following types of questions were difficult to answer: questions about how care could be improved, requests to identify which clinician was primarily responsible for a patient’s care, and those asking about communication between health-care teams. A non-HIV specialist physician indicated it was difficult to answer questions about time periods when they were not involved in the patients care. Additional questions suggested by clinicians to be included in questionnaires included a question to identify possible circumstances in which communication between clinicians was difficult (eg, to protect patient confidentiality) and a question about follow-up after the identification of a DDI. Two suggestions were made: shortening the GP questionnaire and presenting the questions in a chronological order.

#### Findings from the independent review panel

Panel members felt that the 20 minutes provided for review of each case was insufficient. Although some felt reducing the amount of cases per panel and allowing more time per case would help, there was agreement that presenting the data differently would also facilitate the review. It was suggested that questionnaires should be sent to all clinicians the patient had seen within the study period, including those from other NHS trusts. Information from the GP was considered to be essential. Copied hospital and GP notes, discharge summaries, and letters were useful.

Panel members suggested making clinical guidelines readily available at the IRP would be helpful. In addition, further questions about prevention was suggested to improve the utility of the questionnaire to the panel. While anonymized copies of hospital and GP records, including discharge summaries and clinical letters, were provided to the IRP, panel members wished to see additional information including laboratory and radiology reports, all patient communication (eg, follow-up communication if a patient did not attend an appointment), and information on psychosocial issues. Panel members also suggested a case summary, including timeline of events, would help facilitate the case review.

### Ratings of the Quality of Care

Individually, IRP participants expressed a lack of confidence in their ratings of overall care due to insufficient time to conduct each review and in light of missing data, particularly from GPs.

Participants indicated that there were insufficient data available to rate care in 30% of occasions (26 of 88 possible ratings). Of those rated, panel members indicated that there was room for improvement in care for most cases, 40 (65%) of 62, although good practice was seen (22 of 62, 35% cases). No panel members indicated that care was less than satisfactory (Table 2), and no harmful practice was identified.

**Table 2.** Rating of Overall Care by Independent Review Panel Members.

	Number of Reviews		Overall Care Rating			
Type of Event (Number of Cases Reviewed)	Number of Reviewers	Number of Cases × the Number of Reviewers	Good Practice, n (%)	Room for Improvement, n (%)	Less Than Satisfactory, n (%)	Insufficient Data to Assess Quality, n (%)
CKD (2)	8	16	2 (29)	5 (71)	0	9 (56)
DDI (3)	8	24	1 (5)	19 (95)	0	4 (17)
MI (3)	8	24	9 (53)	8 (47)	0	7 (29)
Stroke (3)	8	24	10 (56)	8 (44)	0	6 (25)
Total (11)	8	88	22 (35)	40 (65)	0 (0)	26 (30)

Abbreviations: CKD, chronic kidney disease progression to stage 3; DDI, drug–drug interaction; MI, myocardial infarction.

## Discussion

Our findings demonstrate that this methodology met some, but not all, of the predefined outcomes for feasibility. Domains that were considered feasible include patient participation, questionnaire completion by secondary care physicians, and quality of completed questionnaire data. Clinical questionnaires were returned by approximately two-thirds of secondary care physicians (HIV and non-HIV specialists); only a third of GPs returned completed questionnaires. This will require substantial modification to the method. Similarly, further adaptation is required to the questionnaire process and data provision to the IRP.

The questionnaire return rate for this pilot is lower than 3 most recent return rates reported by NCEPOD (range: 80%-86%).^30-33^ Response of NCEPOD questionnaires may be enhanced by the NCEPOD policy of identifying a named contact (the NCEPOD local reporter) at each hospital who acts as a link between NCEPOD and hospital staff in order to facilitate data collection and return. General practitioners were required to provide greater amounts of data than their secondary care colleagues, as the research team was unable to assist them in the extraction of information from the primary care electronic health records, whereas, for secondary care clinicians, the research team had access to hospital records and so the questionnaire was shorter.

General practitioners reported that they had insufficient time for research tasks, and some requested financial compensation for their time. In the future, we need to consider financial incentives for completed questionnaires. In addition, if the research team were able to obtain access to the primary care databases to extract data as they did for hospital records, the length of GP questionnaires could be reduced. Although patients, hospital physicians, pharmacists, and GPs contributed to the study design, our experience indicates that further patient involvement and consultation with GPs will be required to refine the methodology to enhance GP involvement and optimize return rates.

The creation of the data bundles, including anonymized medical records, was valued by IRP members and however was extremely time intense for the research team. The quality of data returned was high, with a median of 100% questionnaire items completed. Feedback from clinicians indicated that the areas that they had most difficulty answering were questions regarding the communication between various teams and determining which team had responsibility for managing the patient. These are areas of particular interest in the prevention and management of comorbidity, since it has been recognized previously that problems in communication between teams can be problematic as has previously been reported in studies of patients with multimorbidity.^34-36^ Additional questionnaire content may be generated by literature review and qualitative research. By investigating 4 different serious medical events, the lessons learnt are more general, such as communication, as the causative factors for each medical event may differ. More specific lessons could be learnt by investigating 1 type of event alone, such as DDI.

Feedback from the IRP highlighted several areas in which our methodology needs to be improved, including greater time allocated for the review, the collection and presentation of additional information, the inclusion of a patient summary, and a timeline of events. Missing data were flagged as a particular problem when attempting to assess the quality of care. These findings emphasize the need for significant modifications to the methodology in order to optimize completion and return rates, and in order to assess the quality of care, before embarking on a larger study.

## Conclusions

In our small sample, we found that the method was acceptable to patients, and there were satisfactory return rates from secondary care physicians. Further work is needed to refine the methodology in order to increase data returns from GPs and to facilitate the independent panel review.
